# Connectivity in a pond system influences migration and genetic structure in threespine stickleback

**DOI:** 10.1002/ece3.476

**Published:** 2013-01-18

**Authors:** Mathew Seymour, Katja Räsänen, Rolf Holderegger, Bjarni K Kristjánsson

**Affiliations:** 1 Department of Aquatic Ecology and Institute of Integrative Biology, EAWAGETH Zurich, Überlandstrasse 133, CH-8600 Dübendorf, Switzerland; 2 Department of Aquaculture and Fish Biology, Hólar University CollegeHáeyri 1, 550 Skagafjörður, Iceland; 3 Biodiversity and Conservation Biology, WSL Swiss Federal Research InstituteCH-8903 Birmensdorf, Switzerland

**Keywords:** *Gasterosteus aculeatus*, Iceland, landscape genetics, microsatellites, periodic flooding

## Abstract

Neutral genetic structure of natural populations is primarily influenced by migration (the movement of individuals and, subsequently, their genes) and drift (the statistical chance of losing genetic diversity over time). Migration between populations is influenced by several factors, including individual behavior, physical barriers, and environmental heterogeneity among populations. However, drift is expected to be stronger in populations with low immigration rate and small effective population size. With the technological advancement in geological information systems and spatial analysis tools, landscape genetics now allows the development of realistic migration models and increased insight to important processes influencing diversity of natural populations. In this study, we investigated the relationship between landscape connectivity and genetic distance of threespine stickleback (*Gasterosteus aculeatus*) inhabiting a pond complex in Belgjarskógur, Northeast Iceland. We used two landscape genetic approaches (i.e., least-cost-path and isolation-by-resistance) and asked whether gene flow, as measured by genetic distance, was more strongly associated with Euclidean distance (isolation-by-distance) or with landscape connectivity provided by areas prone to flooding (as indicated by *Carex* sp. cover)? We found substantial genetic structure across the study area, with pairwise genetic distances among populations (D_PS_) ranging from 0.118 to 0.488. Genetic distances among populations were more strongly correlated with least-cost-path and isolation-by-resistance than with Euclidean distance, whereas the relative contribution of isolation-by-resistance and Euclidian distance could not be disentangled. These results indicate that migration among stickleback populations occurs via periodically flooded areas. Overall, this study highlights the importance of transient landscape elements influencing migration and genetic structure of populations at small spatial scales.

## Introduction

Much evidence exists on how natural selection and mutations shape population structure (Darwin 1859; Coyne and Orr 2004; Schluter 2009; Roesti et al. 2012). However, neutral processes such as migration and drift are also important (Hartl and Clark 1997; Neiva et al. 2012). Considering a single population, the extent of neutral genetic diversity over time is expected to be mainly related to its effective population size, the amount of migration to and from the population, and genetic drift (Slatkin 1987). If migration between populations is high, the amount of genetic diversity within populations is expected to be greater compared with populations with limited or no migration. The further apart two populations are in space, the less likely migration between the two populations will occur, thereby increasing their genetic differentiation due to isolation-by-distance (IBD) (Wright 1951). IBD is, however, an overly simplistic descriptor of differentiation, because it ignores varying landscape elements between populations, which may also affect migration. With the recent increase in geospatial information, it is now possible to assess realistic migration pathways based on landscape connectivity using landscape genetic approaches (Manel et al. 2003; Zeller et al. 2012). Many landscape genetic studies have indicated the importance of landscape connectivity for migration between populations, thereby influencing the spatial genetic structure of populations (Manel et al. 2003; Storfer et al. 2010). Primarily, persistent landscape elements, such as natural barriers, anthropogenic development, or rivers (e.g., Spear et al. 2005; Leclerc et al. 2008; Raeymaekers et al. 2008), have been used to infer discrete barriers to gene flow. Transient landscape elements, such as areas related to fire and flooding, have received considerably less attention, but have also shown to be important in regional genetic structure of species (Kitamoto et al. 2005; Murphy et al. 2010). Here, we assess the influence of transient landscape elements on threespine stickleback (*Gasterosteus aculeatus*) inhabiting a series of ponds in Belgjarskógur, Northeast Iceland.

Threespine stickleback is a common and short-lived (up to 2–3 years) fish species, which marine ancestors have repeatedly colonized a wide range of freshwater environments across the northern hemisphere since the last ice age (<14,000 ybp). Phenotypic divergence of sticklebacks, in response to environmental variation, has often been assessed (Bell and Foster 1994). But, sticklebacks are also well suited for studies assessing the relative influence of selection, migration, or drift on genetic structure, due to a fast reproductive rate, large population sizes, and ability to rapidly colonize new areas (Bell and Foster 1994). For example, Raeymaekers et al. (2008) used the extent of genetic differentiation of sticklebacks as a proxy for connectivity in a Belgian river network. Anthropogenic barriers (e.g., dams) were shown to have greatest impact on gene flow and genetic drift, suggesting strong anthropogenic influences on population genetic structure. Another study, utilizing landscape genetics on stickleback, suggested that salinity facilitates divergence between fresh and saltwater stickleback occurring along the Saint Lawrence river (McCairns and Bernatchez 2008). However, apart from these two studies, migration patterns of stickleback have rarely been studied, especially with respect to the effects of landscape elements. Assessing the influence of landscape heterogeneity on genetic structure of threespine sticklebacks may help to further our understanding of how local populations are connected and general processes influencing microevolution, which are important topics for evolutionary and conservation biology (e.g., Smouse and Peakall 1999; Segelbacher et al. 2010).

**Figure 1:**
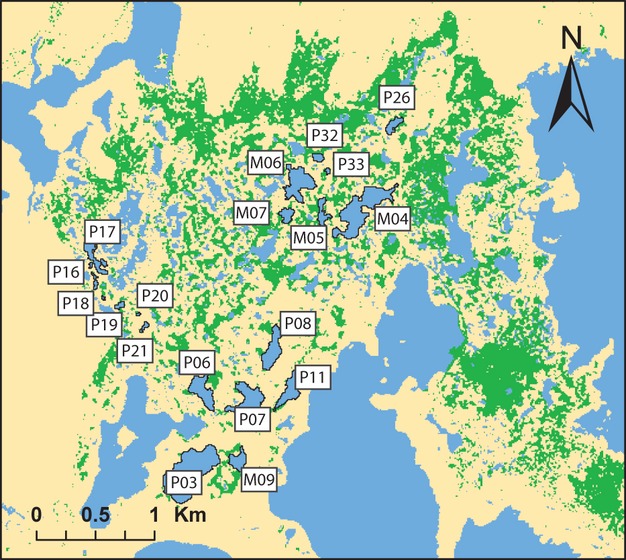
Sampled locations of 19 stickleback ponds (outlined in black), water (blue), *Carex* cover (green), and other land cover types (tan) across the Belgjarskógur pond system in Iceland. The study area is approximately 7.5 km^2^. For population abbreviations, see Table 1.

We investigated the relationship between landscape connectivity and population genetic differentiation in freshwater threespine stickleback across 19 ponds in Belgjarskógur. Belgjarskógur is a small wetland complex (woodland marsh; ∼7.5 km^2^) northwest of Lake Mývatn (Fig. 1) with over 100 small ponds, many of which are inhabited by threespine stickleback. Belgjarskógur is geologically young, formed about 2500 years ago (i.e., about 2000 stickleback generations) after the eruption of Threngslaborgir (Einarsson 1982). Vegetation within Belgjarskógur is characterized by small bushes and sedges, *Carex* sp., specifically *C. rostrata* (Bengtson 1970; Árni Einarsson, pers. comm.). *C. rostrata* is a common colonizer of floodplains and persists across a range of moisture levels (Visser et al. 2000), making it a good indicator of areas experiencing seasonal flooding. Flooding has been shown to influence species richness in plant communities (Ferreira and Stohlgren 1999) and genetic diversity in fish (common roach, *Rutilus rutilus*; Hänfling et al. 2004).

To gain insight to the potential role of seasonal flooding on regional genetic structure, we used two landscape genetic approaches (least-cost-path, LCP, and isolation-by-resistance, IBR) across 19 stickleback ponds in Belgjarskógur. We investigated the following main questions: (1) What is the extent of genetic structure of stickleback populations in Belgjarskógur? (2) Is genetic distance (D_PS_) among population pairs simply correlated with Euclidean distance (IBD) or is it more related to landscape connectivity provided by areas prone to flooding?

## Material and Methods

### Sampling and genotyping

Freshwater sticklebacks (*Gasterosteus aculeatus* subsp. *leiurus*) were collected from 19 ponds across Belgjarskógur during June 2009 (Fig. 1) using unbaited minnow traps laid overnight. Sampling was repeated daily until at least 30 adult size sticklebacks (>30 mm in length) were caught per pond. Sticklebacks were euthanized using an overdose of phenoxyethanol. From each individual, pectoral fin clips were taken and preserved in 95% ethanol for later genetic analyses. The total data set comprised 454 individuals (Table 1).

**Table 1:** Pond identity (Pond), number of individuals genotyped (N), allelic richness (Ar), expected heterozygosity (He), observed heterozygosity (Ho), fixation index (F_IS_) and its 95% confidence intervals (F_IS_ CI), *P*-value for deviation from Hardy–Weinberg equilibrium (HWE), estimate of effective population size (Ne) and its 95% confidence intervals (Ne CI), and identification of loci suspected of null alleles (Null) in stickleback from 19 ponds from the Belgjarskógur pond system in Iceland (total number of individuals studied = 454)

Pond	N	Ar	He	Ho	F_IS_	F_IS_CI	HWE	Ne	NeCI	Null
M4	22	3.87	0.52	0.53	0.000	(−0.103–0.035)	0.313	45	(34–103)	N/A
M5	21	3.94	0.50	0.44	0.110	(−0.030–0.166)	0.065	38	(24–96)	174
M6	23	4.04	0.52	0.47	0.089	(−0.014–0.143)	0.107	35	(31–134)	N/A
M7	18	3.95	0.50	0.44	0.115	(−0.041–0.205)	0.298	23	(24–82)	7033
M9	28	3.29	0.41	0.40	0.042	(−0.076–0.127)	0.041	23	(16–53)	N/A
P3	26	3.39	0.44	0.39	0.108	(0.012–0.169)	0.015	42	(28–137)	4170 & 1125
P6	25	3.34	0.47	0.45	0.060	(−0.077–0.154)	0.120	32	(23–71)	N/A
P7	22	3.64	0.47	0.45	0.053	(−0.079–0.130)	0.138	37	(27–86)	30
P8	26	3.36	0.47	0.47	0.000	(−0.145–0.076)	0.636	45	(34–91)	N/A
Pll	26	3.32	0.50	0.48	0.042	(−0.062–0.106)	0.100	37	(27–88)	N/A
P16	25	3.73	0.47	0.46	0.017	(−0.098–0.084)	0.110	36	(27–70)	N/A
P17	25	3.35	0.43	0.40	0.051	(−0.066–0.132)	0.089	49	(33–135)	N/A
P18	25	2.80	0.32	0.29	0.096	(−0.065–0.217)	0.015	56	(36–218)	N/A
P19	24	2.89	0.37	0.31	0.158	(0.012–0.249)	0.043	86	(45–375)	130
P20	21	2.81	0.38	0.36	0.043	(−0.098–0.128)	0.060	30	(22–68)	N/A
P21	20	2.70	0.43	0.40	0.056	(−0.129–0.179)	0.016	12	(9–21)	174 & 130
P26	28	3.28	0.45	0.46	0.000	(−0.162–0.075)	0.076	48	(31–129)	N/A
P32	24	3.78	0.51	0.50	0.015	(−0.086–0.074)	0.291	45	(33–88)	N/A
P33	25	3.74	0.50	0.52	0.000	(−0.160–0.004)	0.495	40	(30–79)	N/A

DNA was isolated using a high salt extraction method (Aljanabi and Martinez 1997). Neutral genetic structure for each population was estimated using ten hypervariable microsatellite loci, retained from a set of 12 loci (Gac1097, Gac1125, Gac5196, Gac4170, Gac7033, STN30, Gaest66, STN173, STN196, STN70, STN174, STN130; Largiader et al. 1999; Peichel et al. 2001; Mäkinen and Merilä 2008). Polymerase chain reactions (PCR), as detailed in Raeymaekers et al. (2008), were run in two multiplex reactions on a Biometra T1 Thermocycler: multiplex 1 (multiplex 1 Gac1097, Gac1125, Gac5196, Gac4170, Gac7033; 95°C for 15 min, 30 cycles of 94°C for 30 sec, 55°C for 90 sec, 72°C for 10 min; multiplex 2 STN30, Gaest66, STN173, STN196, STN70, STN174, STN130; 95°C for 15 min, 26 cycles of 94°C for 30 sec, 53°C for 90 sec, 72°C for 10 min and 60°C for 30 min; Raeymaekers et al. 2008). PCR products were diluted 1:10 and run on a 3730xl automated sequencer (Applied Biosystems, Carlsbad, CA) using Liz500 as size standard. Alleles were scored using PEAKSCANNER (Applied Biosystems).

### Genetic analysis

Expected (H_e_) and observed (H_o_) heterozygosity as well as allelic richness (Ar) were calculated using FSTAT 2.9.3 (Goudet 1995). All loci were checked for null alleles (Pemberton et al. 1995) using MICROCHECKER 2.2.3 (Van Oosterhout et al. 2004). Null alleles appeared to be present in stickleback from six ponds at five different loci (Table 1). Adjusted allelic frequencies were determined for each locus and pond potentially containing null alleles using BROOKFIELD ESTIMATOR 2 (Brookfield 1996). We then calculated genetic distances among ponds using D_PS_ (see below) for both adjusted and unadjusted allele frequencies. However, as the results for adjusted allele frequencies were quantitatively similar (Pearson *r* = 0.999), we only present the results for unadjusted allele frequencies here. Markers were tested for linkage disequilibrium using FSTAT (Goudet 1995), and deviations from Hardy–Weinberg equilibrium (HWE; Louis and Dempster 1987) were assessed using the exact test implemented in GENEPOP 4.0 (Raymond and Rousset 1995). Within population, fixation indices (F_IS_; Wright 1951) and corresponding confidence intervals were calculated using GENETIX 4.05 (Belkhir et al. 2000).

Pairwise genetic distances between each pair of ponds were calculated as the proportion of shared alleles (D_PS_) (Bowcock et al. 1994) using MICROSAT 1.5 (Minch 1997). The proportion of shared alleles has been found to be more reliable than measurements based on genetic differentiation, such as F_ST,_ in assessing among population genetic distances of closely related populations (Bowcock et al. 1994; Takezaki and Nei 1996). Moreover, as pairwise F_ST_ values calculated in FSTAT (Goudet 1995) were highly correlated with pairwise D_PS_ values (Pearson *r* = 0.815), only results based on D_PS_ are presented here. Effective population sizes (N_e_) and their corresponding 95% confidence intervals were calculated via approximate Bayesian computation using the program ONeSAMP 1.2 (Tallmon et al. 2008). Effective population size represents the number of individuals in an ideal population with the same rate of genetic drift as in the actual population (Futuyma 1997).

Population genetic structure across the study area was assessed using Bayesian clustering in STRUCTURE with the admixture model (Pritchard et al. 2000). STRUCTURE utilizes a model-based method to create a user-specified number of clusters and assigns individuals to these clusters based on their multilocus genotypes, without prior knowledge of sampling locations. We tested for one to 20 clusters (*K* = 1–20) using 10 runs per *K*, 10,000 burn in and an additional 100,000 Markov chains to determine the overall log probability of the tested value of *K*. The best *K* was identified using the method of Evanno et al. (2005).

Previous studies indicated that three of the microsatellite loci used here could be linked to quantitative trait loci (QTL) including dorsal spine length (Gac7033 and STN130), lateral plate width and height (Gac1125), and short gill raker number (STN130; Peichel et al. 2001; Mäkinen et al. 2008). We thus performed a series of tests to investigate whether these potentially QTL-linked loci behaved in a neutral way in this study. First, we checked for locus-wise deviations from HWE for each of the three loci separately, but did not find any. Second, we reran the STRUCTURE analysis, but the major groupings inferred stayed qualitatively constant with respect to the number of clusters, cluster assignment, and geographic patterns of the inferred clusters, when the above three loci were removed one after the other compared with when all loci were included. When all three loci were removed, we found one cluster less (*K* = 5 instead of *K* = 6; see results section), but this was likely due to a drop in the number of alleles from 95 with all loci included to 56 when the above three loci were removed. Third, we recalculated pairwise D_PS_ by removing each of the potentially QTL-linked loci separately as well as all three together from the data set. We then checked for correlations between these and the pairwise D_PS_ values from the whole data. We found high positive correlations (Fig. S1). All the above findings show only limited influence of the potentially QTL-linked loci on the results of this study.

### Landscape genetic analysis

The flora of Belgjarskógur is dominated by bushes and sedges (*Carex* sp.), with *C. rostrata* dominating the sedge communities (Bengtson 1970; Árni Einarsson, pers. comm.). *C. rostrata* is specifically known for establishing and persisting in seasonally flooded areas, making it a potentially suitable proxy for identifying regions prone to seasonal flooding (Visser et al. 2000). To test whether threespine stickleback migration in Belgjarskógur is influenced by periodic flooding events or primarily by Euclidean distance, we quantified landscape connectivity based on presence of *Carex* sp. (henceforth, *Carex* cover) and Euclidean distance. To determine *Carex* cover across Belgjarskógur, we first used high-resolution (2.5-m pan-sharpened spatial resolution) SPOT-5 satellite imagery obtained from the National Land Survey of Iceland for the years 2002/2003. Using ARCGIS 10 (ESRI), we then created a raster grid (resistance surface) (10 × 10 m) of Belgjarskógur, using the maximum-likelihood classification function to classify areas to either “water,” “*Carex* cover,” or “other ground cover” landscape element classes. These three classes were preidentified using five training samples for each landscape element class based on GPS-referenced locations across Belgjarskógur (Campbell 2002).

Following Zeller et al. (2012), we used a two-stage empirical approach to systematically explore the parameter space of the resistance surface scenarios with respect to *Carex* cover. We compared models that differed in resistance values (1, 5, 10, 25, 50, and 100) for *Carex* cover, whereby 1 equals resistance of water (no resistance) and 100 other resistance of ground cover (high resistance). We then used ARCGIS 10 and the ARCTOOLKIT LANDSCAPE (Etherington 2011) to compute least-cost-paths (LCP) between all pairs of ponds for each resistance surface. Additionally, we also computed Euclidean distance between all pairs of ponds by setting all raster cells to a resistance value of 1. As an alternative to LCP between each pair of ponds, we also calculated distances between ponds using isolation-by-resistance (IBR) as implemented in CIRCUITSCAPE, which calculates all potential resistance pathways between each pair of ponds and is based on electrical current theory (McRae 2006).

To determine whether LCP, IBR, or Euclidean distance had a greater influence on genetic distance, we used Mantel and partial Mantel tests in a causal modeling framework, following Cushman et al. (2006). Mantel tests (Mantel 1967) with 10,000 permutations were used to determine the significance of correlations between matrices of pairwise LCP/IBR and genetic distances (D_PS_). Partial Mantel tests with 10,000 permutations were used to separate the effects of LCP/IBR from those of Euclidean distance alone. All Mantel and partial Mantel tests were calculated using the Vegan package (Oksanen et al. 2009) in R (Team 2012). Despite the debate regarding the statistical properties of partial Mantel tests (Castellano and Balletto 2002), Legendre and Fortin (2010) showed that they work well in simple cases using true distance matrices, such as in this study.

## Results

### Genetic Variation

Sticklebacks in five ponds showed significant deviations from HWE (*P* ≤ 0.05), suggesting they might be influenced by inbreeding or nonrandom mating (Table 1). F_IS_ values ranged from 0.00 to 0.16 across the ponds, but only in two ponds did F_IS_ values significantly deviate from zero further suggesting inbreeding or nonrandom mating (Table 1). Overall, F_ST_ values were not strongly affected when we included or excluded stickleback in the five ponds deviating from HWE (F_ST_ = 0.082–0.090 when single ponds were removed; F_ST_ = 0.084 when all ponds were included). Additionally, STRUCTURE analyses without these five ponds showed similar geographic clustering. In conclusion, we present the data including all stickleback from the 19 ponds below.

**Figure 2:**
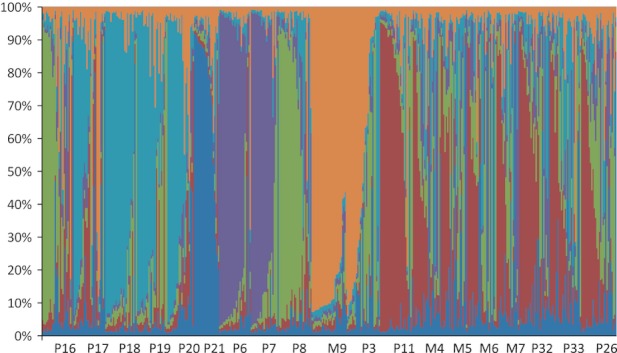
STRUCTURE analysis showing the most likely clustering with *K* = 6. Each bar represents a sampled individual stickleback, and colors represent particular genetic clusters. Percent likelihood of an individual belonging to a given cluster is indicated on the *y*-axis, and the populations where individual where sampled are given on the *x*-axis.

Average allelic richness (Ar) across loci ranged between 2.70 and 4.04 (Table 1) and effective population size (N_e_) ranged from 12 to 86. Pairwise D_PS_ values among ponds ranged from 0.118 to 0.488. STRUCTURE analysis indicated six genetic clusters (Fig. 2). Sticklebacks in the seven eastern ponds (M4, M5, M6, M7, P32, P33, and P26) were not assigned to specific genetic groups and appeared to be a set of highly admixed populations. In contrast, sticklebacks in the western ponds formed six genetic groups (first group: P16, P17, P18, and P20, second: P6 and P7; third: P21; fourth: P8 and P16, fifth: M9 and P3, and sixth: P11).

### Landscape genetic analysis

There were significant positive correlations between genetic distances, as measured by D_PS_ and all LCP and IBR distance measurements (Tables 2, 3). For all resistance values of *Carex* cover, except for values 50 and 100, D_PS_ was more strongly correlated with LCP and IBR than with Euclidean distances (Tables 2, 3). With increasing resistance values for *Carex* cover, both LCP and IBR analyses showed decreasing correlations with genetic distances (Tables 2, 3).

**Table 2:** Results of Mantel and partial Mantel tests (Mantel *r* and *P*-Value) of genetic distances (D_PS_) among 19 stickleback populations from the Belgjarskógur pond system, Iceland, and least-cost-path (LCP) and Euclidean distances regarding different resistance values (*Carex* sp. cover resistance set as 1 = low resistance to 100 = high resistance)

			Partial Mantel test			
			
Mantel test			Euclid | LCP		LCP | Euclid	
Resistance	Mantel *r*	*P*-value	Mantel *r*	*P*-value	Mantel *r*	*P*-value
Euclidean	0.140	<0.01				
1	0.271	<0.01	0.053	0.020	0.241	<0.01
5	0.262	<0.01	0.045	0.961	0.224	<0.01
10	0.248	<0.01	0.029	0.884	0.214	<0.01
25	0.217	<0.01	0.027	0.859	0.180	<0.01
50	0.201	<0.01	0.012	0.698	0.153	<0.01
100	0.188	<0.01	0.009	0.354	0.139	<0.01

**Table 3:** Results of Mantel and partial Mantel tests (Mantel *r* and *P*-Value) of genetic distances (D_PS_) among 19 stickleback populations from the Belgjarskógur pond system in Iceland and isolation-by-resistance (IBR) and Euclidean distances regarding different resistance values (i.e., *Carex* sp. cover ranges from 1 = low resistance to 100 = high resistances)

			Partial Mantel test			
			
Mantel test			Euclid | IBR		IBR | Euclid	
Resistance	Mantel *r*	*P*-value	Mantel *r*	*P*-value	Mantel *r*	*P*-value
Euclidean	0.1137	<0.01				
1	0.176	<0.01	0.124	<0.01	0.155	<0.01
5	0.164	<0.01	0.116	<0.01	0.134	<0.01
10	0.149	<0.01	0.115	<0.01	0.115	<0.01
25	0.123	<0.01	0.119	<0.01	0.083	<0.01
50	0.099	<0.01	0.125	<0.01	0.056	0.030
100	0.077	<0.01	0.131	<0.01	0.029	0.181

**Figure 3:**
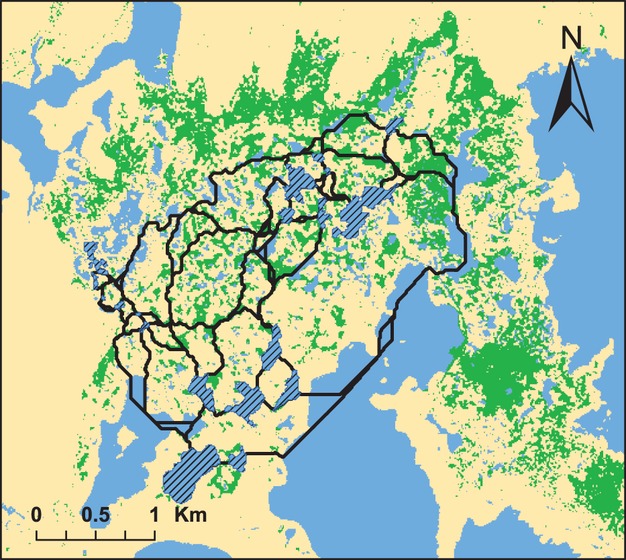
Least-cost-path of landscape connectivity across 19 study ponds (stripped), with the highest correlation with genetic distance (D_PS_) with *Carex* cover (green; resistance = 1), water (blue; resistance =1), and other land cover (tan; resistance = 100) (see Table 2 for analyses).

Partial Mantel tests were used to disentangle the effects of LCP/IBR and Euclidean distances. With LCP, we found a significant relationship between genetic distance and Euclidean distance after the removal of LCP distances with *Carex* cover resistance value of 1, but all others resistance values resulted in nonsignificant results (Table 2). In contrast, all LCP independent of *Carex* cover resistance values were still significantly correlated with D_PS_ after the removal of the effects of Euclidean distance. Additionally, correlations decreased with increasing resistance for *Carex* cover (Table 2). The LCP with the highest correlation was found when *Carex* cover resistance was set to 1 (Fig. 3). In this LCP, only other land cover types exhibited landscape resistance while water and *Carex* cover essentially behaved as geographic distance. The fact that we found significant correlations with *Carex* cover resistance and genetic distances when Euclidean distance was removed, but not when we removed LCP suggested *Carex* cover had a greater influence on the genetic distance than geographic distance.

While IBR was generally less effective than LCP, it still showed significant patterns. All IBR, except where *Carex* cover resistance was 100, were significantly correlated with D_PS_ after the removal of the effects of Euclidean distance. Correlations remained more or less constant across the remaining resistance values of *Carex* cover. Likewise, Euclidean distance after the removal of IBR was still significantly correlated with D_PS_. As with LCP, once Euclidean distance was removed, the IBR with the highest correlation was with a *Carex* cover resistance equaling one. That we did not find a difference in the significance of the correlations when we removed IBR or Euclidean distance suggested that the effects of IBR or Euclidean distance on genetic distances cannot be disentangled using the IBR approach.

## Discussion

We found strong genetic differentiation among stickleback in 19 ponds across Belgjarskógur. First, we found six genetic clusters whereby westernmost ponds showed strong clustering, whereas eastern ponds were highly admixed group of these six genetic clusters. Second, while we found significant isolation-by-distance (IBD) among ponds, least-cost-path (LCP) analyses indicated that genetic structure was more strongly related to *Carex* cover than to geographic distance. This suggested that periodic flooding affects gene flow across stickleback ponds in the study area.

### Genetic differentiation in threespine stickleback

Despite the small spatial extent of the study area, we found strong genetic differentiation of stickleback at loci across ponds with genetic distances (D_PS_) varying from 0.118 to 0.488. These genetic distances are similar to those reported in other stickleback studies (e.g., Reusch et al. 2001; Hendry and Taylor 2004; Leinonen et al. 2006; Ólafsdóttir et al. 2007; Raeymaekers et al. 2007; Berner et al. 2009), even though our study area was much smaller (but see Hendry et al. 2002). In previous studies, strong genetic differentiation was typically linked to secondary colonization or strong ecologically meditated reductions in gene flow. In contrast, in our study system, ecological differences are unlikely the causal agent of the high observed genetic differentiation as ecological and phenotypic differences across the area are subtle (Seymour 2011). Instead, we suggest that genetic structure across the Belgjarskógur study area is more likely driven by variation in spatial connectivity jointly with small local population size. In particular, stickleback inhabiting the easternmost ponds formed a highly admixed group, whereas the westernmost ponds formed six genetically distinct groups. This suggests that there are more barriers to gene flow among the westernmost populations, maybe coupled with past founder events during the colonization of the study area or genetic drift (Mayr 1942), as suggested by the generally low effective population sizes (see below).

In general, populations with small effective population sizes (*N*_e_ < 50) are expected to be under strong influence of genetic drift (Wright 1968; Franklin 1980). In our study, 17 of the 19 study ponds were estimated to have *N*_e_ below 50. It is likely that the small size of the ponds (surface area: 25–520 m^2^) limits local population size, and, hence, genetic drift could be expected to play an important role in shaping genetic structure across the Belgjarskógur area. However, while genetic drift and migration likely contribute to the observed levels of genetic differentiation, migration may predominate over genetic drift in our study system. This is indicated by the significant IBD, and by an even stronger association of genetic distance with periodically flooded areas. However, drift should result in random patterns of genetic differentiation.

Compared with previous stickleback studies (e.g., Raeymaekers et al. 2007; Mäkinen and Merilä 2008), we found relatively low levels of genetic variation (i.e., low allelic richness), and allelic richness was greater in the better connected eastern ponds. In addition, in some ponds fixation indices (F_IS_) increased, potentially pointing to inbreeding or nonrandom mating (Wright 1965; Hartl and Clark 1997). Populations in the less connected western part of Belgjarskógur had somewhat lower observed heterozygosity and higher F_IS_s than populations in the better connected eastern populations. Nonrandom mate choice has been repeatedly found in sticklebacks as a result of ecologically mediated mating isolation (reviewed in Räsänen et al. 2012) and imprinting (Kozak et al. 2011), and has been suggested to be a mechanism to avoid inbreeding (i.e., females select against siblings; Frommen and Bakker 2006). However, given the overall low allelic richness and decreased heterozygosities in the western populations, we suspect that inbreeding (resulting from small local population sizes) rather than nonrandom mate choice was the primary cause of the increased fixation indices there. However, additional research is needed to validate this hypothesis.

### Landscape connectivity

Threespine stickleback is well known for its ability to disperse, as witnessed by frequent and repeated colonization of freshwaters from marine as well as across different freshwater habitats (Bell and Foster 1994). Empirical measurements of freshwater stickleback dispersal show strong habitat fidelity and limited dispersal (Black and Wootton 1970; Bolnick et al. 2009; Moore and Hendry 2009) with males and females showing similar dispersal patterns (Bolnick et al. 2009). Landscape connectivity has been shown to influence neutral genetic differentiation in several other vertebrate species, including fish (e.g., yellow perch, Perca flavescens, Leclerc et al. 2008), amphibians (such as poison dart frogs, *Dendrobates pumilio,* Wang and Summers 2009; moor frogs, *Rana arvalis,* Richter-Boix et al. 2010), and mammals (wolverines, *Gulo gulo,* Schwartz et al. 2009), but its effects have rarely been studied in sticklebacks (but see Raeymaekers et al. 2008; McCairns and Bernatchez 2008). Our LCP analyses indicated that *Carex* cover presents weak, and other land -covers strong, barriers to the movement of sticklebacks. This held true even after partialling out the effects of Euclidean distance. While it is obvious that water provides landscape connectivity in fish, our result also indicates that periodic flooding events increase landscape connectivity. This is evidenced here by correlations between genetic distance of stickleback across ponds and presence of *Carex* sp., which in our study area is primarily *C. rostrata* – a species that prefers waterlogged soils and areas prone to periodic flooding (Busch and Lösc 1999; Visser et al. 2000). Variation in the propensity of periodic flooding may also explain the differences in genetic structure between the northeastern and western ponds: in the highly admixed populations of northeastern Belgjarskógur, flooding seems to occur regularly (*Carex* sp. areas are frequent), while flooding seems to be relatively rare in the highly structured Western populations (*Carex* sp. areas less common). Hence, we suggest that variation in flooding frequency and strength allows for different levels of migration and, thus, gene flow across the study area creating a mosaic of genetic structure across Belgjarskógur.

While we found a significant influence of *Carex* sp. cover on genetic structure when we used LCP, we could not separate between the relative effects of flooding propensity (i.e., *Carex* sp. cover) and Euclidean distance on genetic structure when we used isolation-by-resistance (IBR). This is likely due to CIRCUITSCAPE overestimating the number of pathways between ponds, which will decrease the estimated resistance between the ponds (McRae 2006). This is to be expected as *Carex* sp., while water tolerant, will also persist in areas where flooding is less abundant. With this in mind, we argue that it is more appropriate to use LCP here as sticklebacks are water-bound and will likely migrate through areas with high frequency of flooding, rather than along pathways with any amount of flooding.

## Conclusions

We found that landscape connectivity (through geographic distance and periodic flooding events), jointly with small population size and genetic drift, is an important determinant of neutral genetic structure in Belgjarskógur threespine stickleback. Our study particularly highlights the importance of considering fluctuating landscape features, such as periodic flooding events, in shaping the genetic structure of populations, which otherwise might appear geographically isolated. Such temporarily variable spatial connectivity may be relatively more important at small spatial scales, where the frequency of disturbance is expected to be greater than across larger spatial scale. We therefore suggest studies aiming to understand processes influencing diversity or conservation of local populations, should consider the impacts of temporal variation in landscape features, such as periodic fluctuation or disturbance, and the implications they have on migration.

## Supporting Information

**Figure S1.** Pearson correlations coefficients between pairwise D_PS_ values were calculated by removing three potentially QTL-linked loci (GAC1125, STN130, GAC7033), either separately (A–C) or all three together (D) against values calculated from all microsatellite loci in the data of *Gasterosteus aculeatus*. *R*^2^ values are generally very high when single loci are removed. *R*^2^ values when all three potentially QTL-linked loci were excluded are weaker, probably due to a drop in the number of alleles from 95 (in the whole data set) to 56 (three potentially QTL-linked loci removed).
