# Supplementary figures and images for: Connectivity in a pond system influences migration and genetic structure in threespine stickleback
Source: Ecol Evol. 2013 Jan 18;3(3):492–502. doi: 10.1002/ece3.476 (PMC3605840; doi:10.1002/ece3.476)

D<sub>PS</sub> vs No GAC1125

A

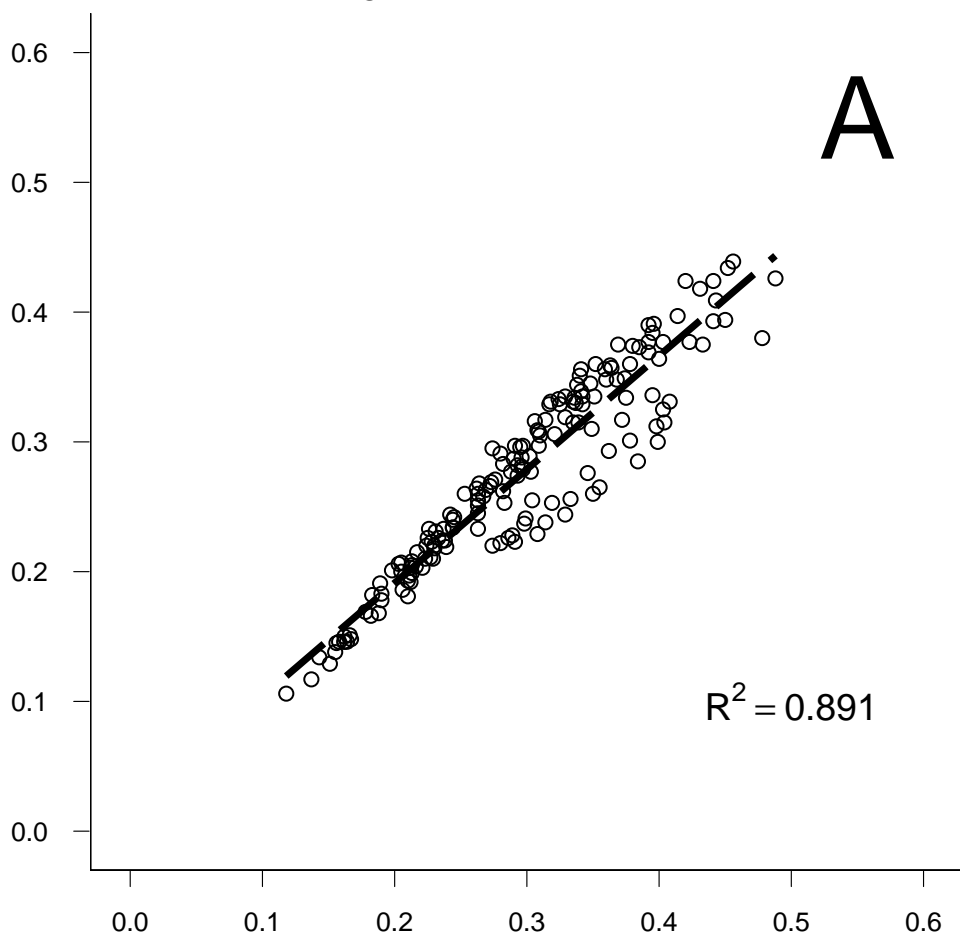

D<sub>PS</sub> vs No GAC1125

B

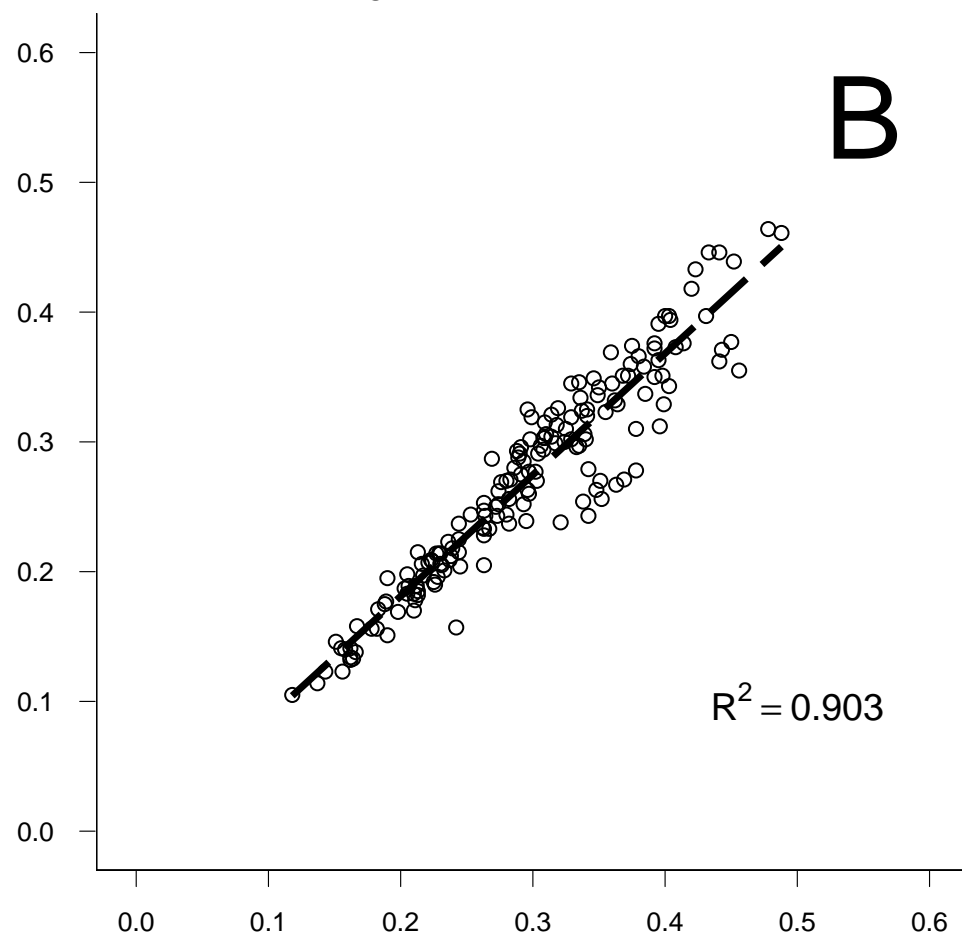

D<sub>PS</sub> vs No GAC1125

C

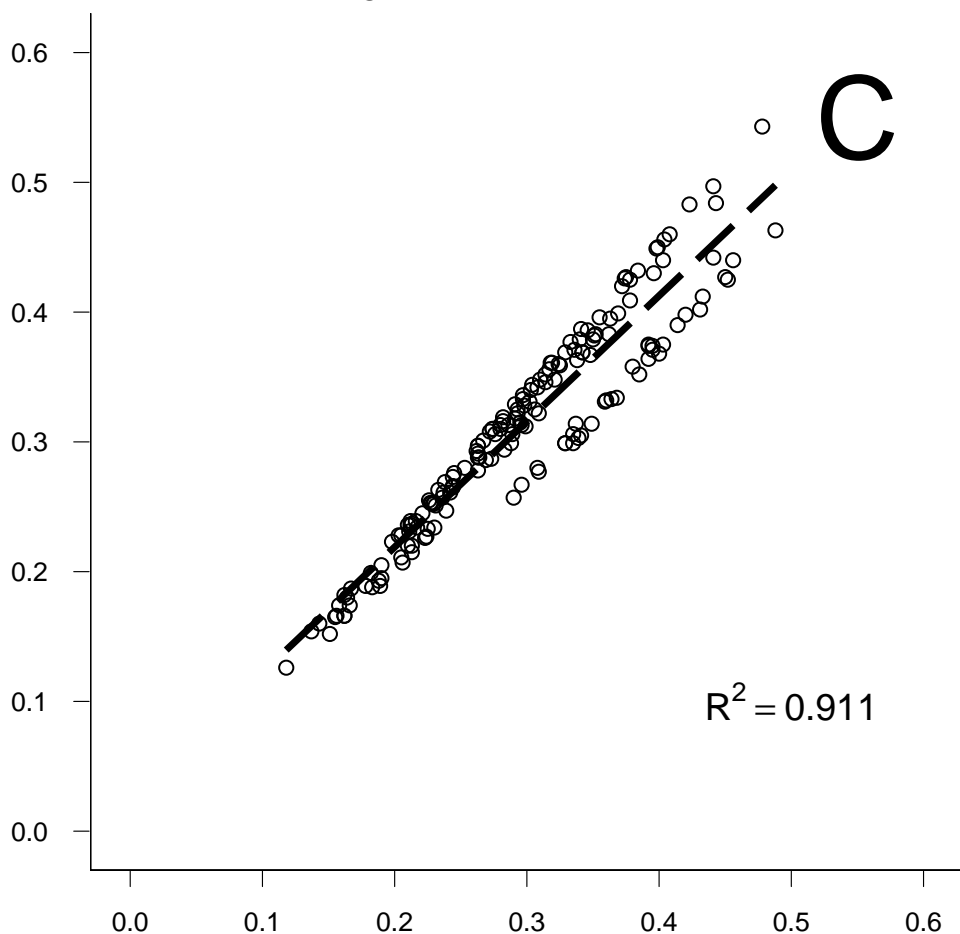

D<sub>PS</sub> vs No GAC1125

D

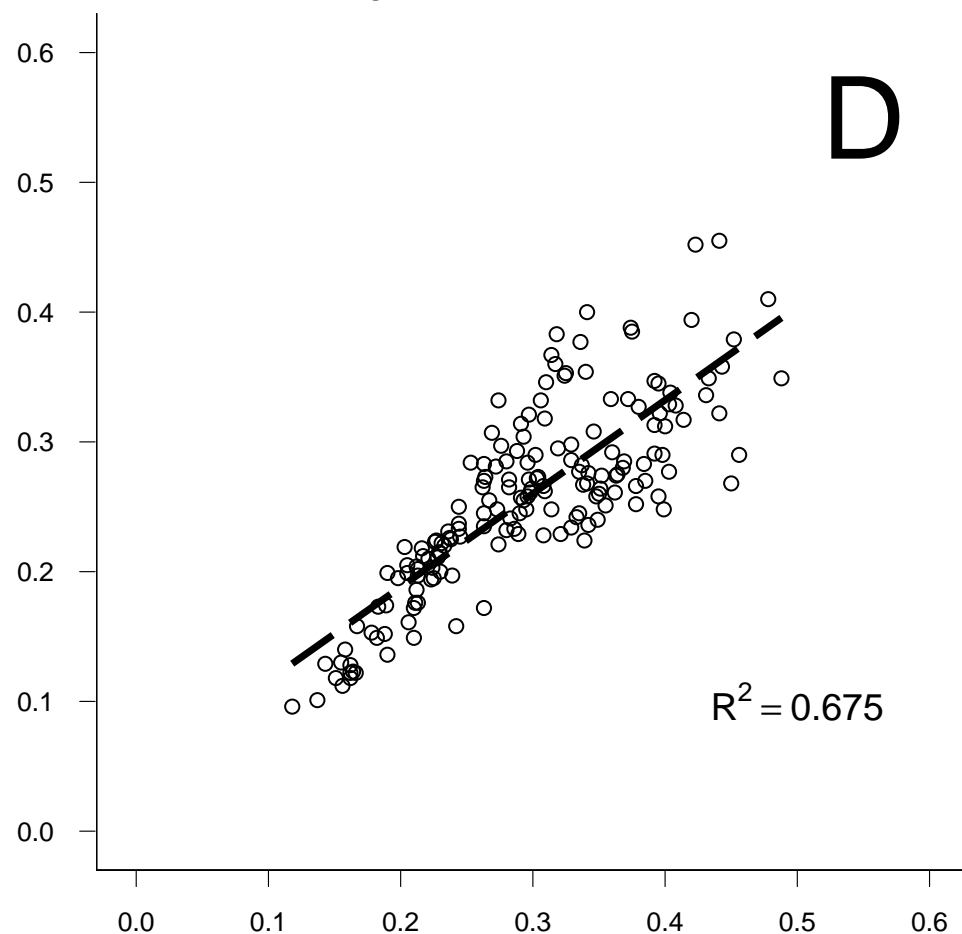

Supplement: Supplementary file 1 [file ece30003-0492-SD1.pdf]
